# Native mass spectrometry reveals the initial binding events of HIV-1 rev to RRE stem II RNA

**DOI:** 10.1038/s41467-020-19144-7

**Published:** 2020-11-13

**Authors:** Eva-Maria Schneeberger, Matthias Halper, Michael Palasser, Sarah Viola Heel, Jovana Vušurović, Raphael Plangger, Michael Juen, Christoph Kreutz, Kathrin Breuker

**Affiliations:** 1 Institute of Organic Chemistry and Center for Molecular Biosciences Innsbruck (CMBI), University of Innsbruck, Innrain 80/82, 6020 Innsbruck, Austria; 2 Present Address: Boston Children’s Hospital, Harvard Medical School, Boston, MA 02115 USA; 3 Present Address: Roche Diagnostics GmbH, 82377 Penzberg, Germany

**Keywords:** RNA-binding proteins, RNA, Mass spectrometry, Retrovirus

## Abstract

Nuclear export complexes composed of rev response element (RRE) ribonucleic acid (RNA) and multiple molecules of rev protein are promising targets for the development of therapeutic strategies against human immunodeficiency virus type 1 (HIV-1), but their assembly remains poorly understood. Using native mass spectrometry, we show here that rev initially binds to the upper stem of RRE IIB, from where it is relayed to binding sites that allow for rev dimerization. The newly discovered binding region implies initial rev recognition by nucleotides that are not part of the internal loop of RRE stem IIB RNA, which was previously identified as the preferred binding region. Our study highlights the unique capability of native mass spectrometry to separately study the binding interfaces of RNA/protein complexes of different stoichiometry, and provides a detailed understanding of the mechanism of RRE/rev association with implications for the rational design of potential drugs against HIV-1 infection.

## Abstract (web summary)

The HIV-1 RNA-binding protein rev facilitates nuclear export of viral RNA. Here, the authors use native mass spectrometry to study the interactions between rev-derived peptides and rev response elements of HIV-1 RNA, providing mechanistic insights into rev recognition and recruitment.

## Introduction

Interactions between proteins and ribonucleic acids (RNA) play important roles in biological processes including retroviral replication^1^. To gain insight into the biochemistry of retroviruses and further antiviral drug development, atomic-level details of RNA-protein complex structures and their binding interfaces can be studied by nuclear magnetic resonance (NMR) spectroscopy or X-ray crystallography. An emerging alternative that can complement these techniques, especially when RNA-protein complexes show unfavorable conformational dynamics that complicate NMR data interpretation^2,3^ or fail to crystallize properly^4,5^, is native top-down mass spectrometry (MS)^6^. We have recently demonstrated that native electrospray ionization (ESI)^7–12^ provides time-resolved, stoichiometric information on complexes of transactivation responsive (TAR) RNA and trans-activating (tat) peptide from human immunodeficiency virus type 1 (HIV-1), and that collisionally activated dissociation (CAD) can reveal tat binding sites of TAR RNA at the single-nucleotide level^6^. This native top-down MS approach relies on the preservation of electrostatic interactions between protonated peptides and deprotonated RNA at energies that are sufficiently high for phosphodiester backbone bond cleavage^6^, for which as few as two arginine residues are sufficient^13^.

While tat binding to TAR RNA increases the production of viral messenger RNA (mRNA)^14^, rev protein binding to rev response element (RRE) segments of unspliced and singly spliced viral mRNA facilitates their nuclear export^15,16^. Both tat and rev are thus critical to the expression of viral proteins and HIV-1 replication^17^. Previous studies have identified nucleotides in stem II of RRE RNA^18–21^ to which the arginine-rich motif (ARM) of rev protein binds^19^ in the assembly of the functional RRE/rev ribonucleoprotein complex in which up to 8–10 rev molecules sequentially associate with a single RRE molecule^22,23^. The N-terminal ARM binding region (residues 34–50, 11 of which are arginine residues) of rev (116 residues) is framed by oligomerization domains (residues 9–26, 51–65) that stabilize the ribonucleoprotein complex^15^. However, as pointed out by Frankel, structural details on the formation of the ribonucleoprotein complex remain largely elusive, and one particularly important question is how the individual subunits of rev recognize the RRE^4^. Likewise, Le Grice stated that the sequence of events required for rev assembly and the positioning of individual rev molecules on the RRE remain unknown, and that the role of the three-way junction from which stem IIB originates in binding of the second rev molecule is unclear^15^. The as yet unresolved challenge to correlate binding regions with individual rev molecules that successively bind to RRE RNA complicates the rational design of highly efficient drugs for the treatment of HIV-1 infection.

**Fig. 1:**
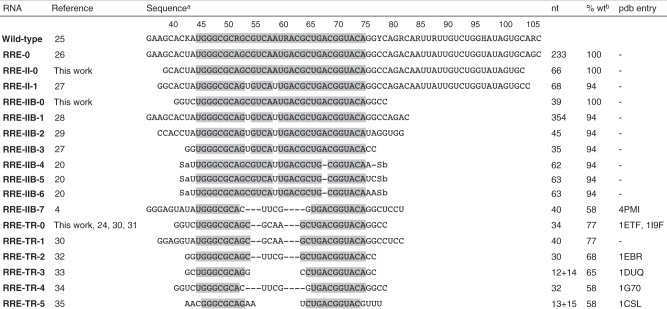
RRE II RNA constructs used in this work (RRE-II-0, RRE-IIB-0, RRE-TR-0) and in previous studies. ^a^From 5′-OH- to 3′-OH-terminus; R = G or A, K = G or U, Y = C or U, H = A, C, or U; Sa and Sb stand for complementary, 15 nt RNA segments designed to form an artificial stem structure^20^ (not counted in %wt), and nucleotides of stem IIB that are conserved between consensus sequences from each of the major families of HIV-1^25^ are highlighted in gray, ^b^sequence similarity to the 45–75 region of wild-type RRE RNA.

Here we study the binding of an ARM peptide derived from rev protein to RRE RNA from HIV-1 by native ESI and CAD MS. Complexes of RRE stem IIB RNA constructs (**RRE-IIB-0**, **RRE-TR-0**, Fig. 1) and full-length RRE stem II RNA (**RRE-II-0**) with rev ARM peptide^24^ are investigated by MS, and the data compared to results from NMR, X-ray crystallography, and biochemical probing experiments^4,20,24–35^. Our ESI and CAD data reveal a mechanism in which rev molecules are sequentially recruited by the upper stem of RRE IIB and relayed to binding sites that allow for rev dimerization rather than directly binding to individual sites as suggested by previous studies.

## Results

### Design of the RRE and rev constructs

The RRE RNA constructs **RRE-II-0**, **RRE-IIB-0**, and **RRE-TR-0** (Fig. 1) instead of full-length RRE RNA (~350 nt) and rev ARM peptide^24^ instead of rev protein (116 residues) were used in this study to limit the mass of the RRE/rev complexes, such that the signals of all fragments from CAD were isotopically resolved on our 7 T FT-ICR mass spectrometer for unambiguous assignment of fragment identity (higher-field FT-ICR instruments have higher mass resolving power, which would allow for the study of larger complexes). Our MS data nevertheless provide meaningful insight into the assembly of the functional RRE/rev ribonucleoprotein complex as the interaction of rev protein with RRE RNA is modular, i.e., rev ARM peptide molecules bind to RRE RNA with the same affinity as rev protein monomer (rev protein with a mutation in its oligomerization domain to prevent rev oligomerization), and requires the same amino acids for recognition^31^. Furthermore, oligomerization-deficient rev protein was shown to assemble on RRE RNA^30,36^ one molecule at a time^22^ to produce complexes with ~12% nuclear export activity compared to those of wild-type rev protein^36^. In the **RRE-IIB-0** RNA construct, A44•G76 of the wild-type RRE RNA was replaced by C44•G76, and two G•C pairs were added for stabilization of the lower stem. The same lower stem was used in the **RRE-TR-0** construct, in which three base pairs (G55•C63, U56•A62, C57•G61) of the upper stem were removed and the wild-type loop was replaced by GCAA.

### Formation of RRE-IIB/rev complexes monitored by native ESI MS

**Fig. 2:**
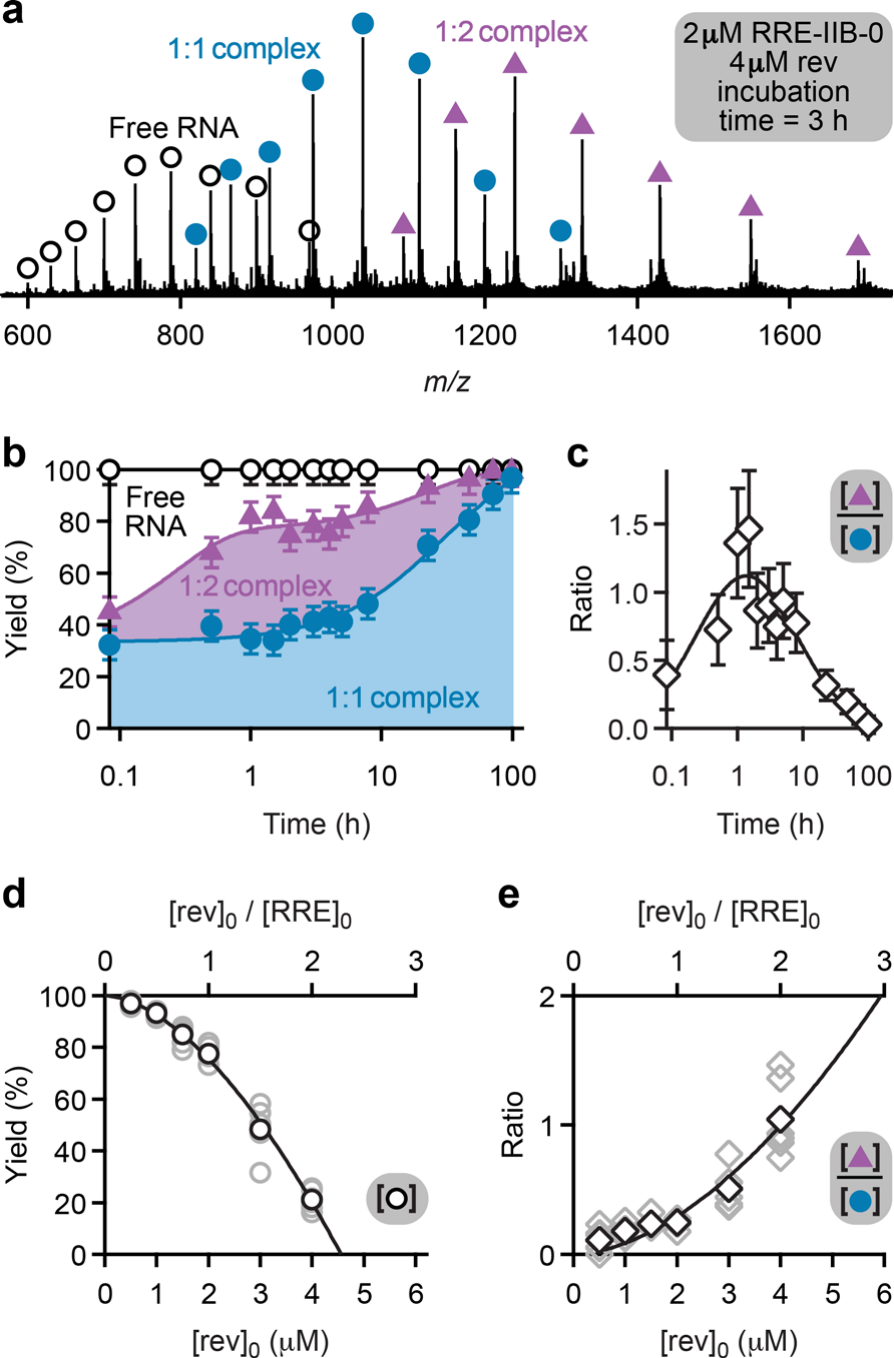
RRE-IIB-0/rev complex stoichiometry probed by native MS. **a** ESI spectrum of a 2 µM **RRE-IIB-0** RNA and 4 µM rev peptide solution in 9:1 H_2_O/CH_3_OH (pH ~7.5, adjusted by addition of ~1.25 mM piperidine; 3 h incubation time) shows signals of free RNA (open circles), 1:1 complex (filled circles), and 1:2 complex (triangles); **b** stacked area plot illustrating the yield of free RNA (open circles), 1:1 complex (filled circles), and 1:2 complex (triangles), error bars show estimates obtained as described in the Methods section; **c** Ratio of 1:2 to 1:1 complex yields (diamonds) versus incubation time with error bars as described in the Methods section; **d** yield of free RNA at *t* = 1–5 h (gray circles, average values shown as black circles) and **e** ratio of 1:2 to 1:1 complex yields at *t* = 1–5 h (gray diamonds, average values shown as black diamonds) versus rev peptide concentration, [rev]_0_ (**RRE-IIB-0** concentration, [RRE]_0_, was 2 µM in all experiments). Source data are provided as a Source Data file.

The spectrum in Fig. 2a, recorded 3 h after preparation of a solution with **RRE-IIB-0** RNA (2 µM) and rev ARM peptide (4 µM) for ESI MS (i.e., 3 h incubation time), shows signals of free RNA (~22%), 1:1 RNA-peptide complexes (~41%), and 1:2 RNA-peptide complexes (~37%). The 1:1 complexes must have formed at a relatively high rate as their yield was already ~32% at 5 min incubation time (Fig. 2b). A far slower increase in yield of 1:1 complexes (<2% h^−1^) was observed during the next ~8 h, and a yield of ~97% was only reached at 99 h incubation time (Fig. 2b). By contrast, the yield of 1:2 complexes increased from ~13% at 5 min to ~50% at 1 h, and then slowly decreased to <3% at 99 h such that the ratio of 1:2 to 1:1 complex yields was close to unity (0.97 ± 0.28; average ± standard deviation) in the period 0.5–8 h (Fig. 2c). These data are evidence for highly intricate kinetics for the association and dissociation of 1:1 and 1:2 complexes of **RRE-IIB-0** RNA and rev ARM peptide, show that the reaction RRE•rev + rev → rev•RRE•rev is reversible, and confirm previous findings of sequential rev binding to RRE RNA^11,22^. Importantly, the ESI data reveal that RRE stem IIB RNA can bind two rev ARM peptides, even at a peptide (0.5 µM) to RNA (2 µM) concentration ratio ([rev]_0_/[RRE]_0_) of 0.25 that limited overall complex formation to ~3% (~97% free RNA, Fig. 2d). With increasing rev peptide concentration [rev]_0_, the yield of free RNA decreased (Fig. 2d), and not only the yield of complexes but also the ratio of 1:2 to 1:1 complex yields increased for incubation times between 1 and 5 h (Fig. 2e and Supplementary Fig. 2). To investigate the dynamics of rev association and dissociation, we monitored the yields of free RNA and 1:1 and 1:2 RNA-peptide complexes after addition of rev ARM peptide to a solution of **RRE-IIB-0** RNA and isotopically labeled rev ARM peptide, rev* (which is 30.0249 Da higher in mass than rev), on a time scale of up to 2700 s (see Supplementary Fig. 3). The yield data were best fit with double exponential functions that indicate a slow and a fast process whose rates differed by a factor of ~9 (see Supplementary Table 1). The change in the fractions of 1:1 complexes with rev and rev* attached (see Supplementary Fig. 3c) was best fit with single exponential functions at rates that were, within error limits, the same as those of the fast process (see Supplementary Table 1). We conclude that the fast process (~0.007 s^−1^) reflects the formation of 1:1 complexes and the slow process (~0.0008 s^−1^) reflects the formation of 1:2 complexes, in agreement with the data in Fig. 2b. The 1:2 complexes were observed with 2·rev, rev+rev*, and 2·rev* attached, whose fractions (see Supplementary Fig. 3d) were best fit with single exponential functions at rates (~0.013 s^−1^) that were ~17-fold higher than the rates for the formation of 1:2 complexes (see Supplementary Table 1). These findings show that the binding of rev ARM peptide to **RRE-IIB-0** RNA is a highly dynamic process in which rev exchanges at a rate that exceeds the observed rate of complex formation, consistent with single-molecule fluorescence spectroscopy studies by Millar and coworkers^22^.

The data in Fig. 2d were empirically found to fit a power function, *f*([rev]_0_) = 100–7.5 ± 0.7·[rev]_0_^1.71^^ ± 0.08^ (±values are standard deviations of the fit coefficients), that extrapolates to 0% free RNA at [rev]_0_/[RRE]_0_ ~2.3. Likewise, the data in Fig. 2e were found to fit a power function, *f*([rev]_0_) = 0.086 ± 0.033·[rev]_0_^1.8 ± 0.3^, that extrapolates to a ratio of 1:2 to 1:1 complex yields of ~2 at [rev]_0_/[RRE]_0_ = 3. We conclude that at least for incubation times between 1 and 5 h, 1:2 RRE/rev complexes constitute the major fraction of species in solutions of RRE and rev at concentrations that allow for binding of all RRE. Complexes of RNA constructs that resemble RRE stem IIB (e.g., RNAs **RRE-IIB-2**, **RRE-IIB-4**, **RRE-IIB-5**, **RRE-IIB-6**, **RRE-IIB-7**, and **RRE-TR-1**, Fig. 1) and rev protein or rev peptides in a 1:2 ratio were also observed in gel shift assay studies^4,20,29,30^ and by X-ray crystallography^4^. RRE and rev concentrations in HIV-1 infected cells are not known, but because protein-to-mRNA ratios are typically in the 10^2^–10^4^ range^37^, binding of 2 rev molecules to stem IIB of RRE in cells should be even faster than in the experiments here. Furthermore, the yield of 1:2 RRE/rev complexes should be higher with wild-type rev protein than in our study with rev ARM peptide as wild-type rev protein binds more strongly (*K*_D_ = 0.12 ± 0.00062 nM) to RRE RNA than both oligomerization-deficient rev protein (*K*_D_ = 15 ± 1.7 nM) and rev ARM peptide (*K*_D_ = 13 ± 2.0 nM).

### Binding site mapping of RRE IIB/rev complexes by CAD MS

We next studied the sites of binding of rev ARM peptide to **RRE-IIB-0** RNA (Fig. 1) by CAD MS. Mass spectrometry is unique in that complexes of differing stoichiometry can be separately isolated in the mass spectrometer for further investigation, provided that their mass-to-charge (*m*/*z*) values do not overlap. As shown in Fig. 2a, the *m*/*z* values of (RNA + m·peptide - nH)^n−^ ions with *m* = 0, 1, and 2 were well separated at all *m* and *n* values. For binding site mapping in the 1:1 and 1:2 complexes by CAD, we isolated (**RRE-IIB-0** + 1·rev - 15H)^15−^ ions at *m*/*z* ~1040 and (**RRE-IIB-0** + 2·rev - 16H)^16−^ ions at *m*/*z* ~1160, respectively. These ions were selected because of their high abundance (Fig. 2a) and because their net negative charge per nucleotide (~0.4 charges/nt) was similar to that used for binding site mapping in TAR/tat complexes^6^. From site-specific yields of ***c*** and ***y*** fragments from phosphodiester backbone bond cleavage by CAD^38–40^, with and without one or two peptides attached (see Supplementary Fig. 4), we calculated site-specific occupancy values that, when plotted versus the cleavage site of the RNA, reveal peptide binding regions^6^.

**Fig. 3:**
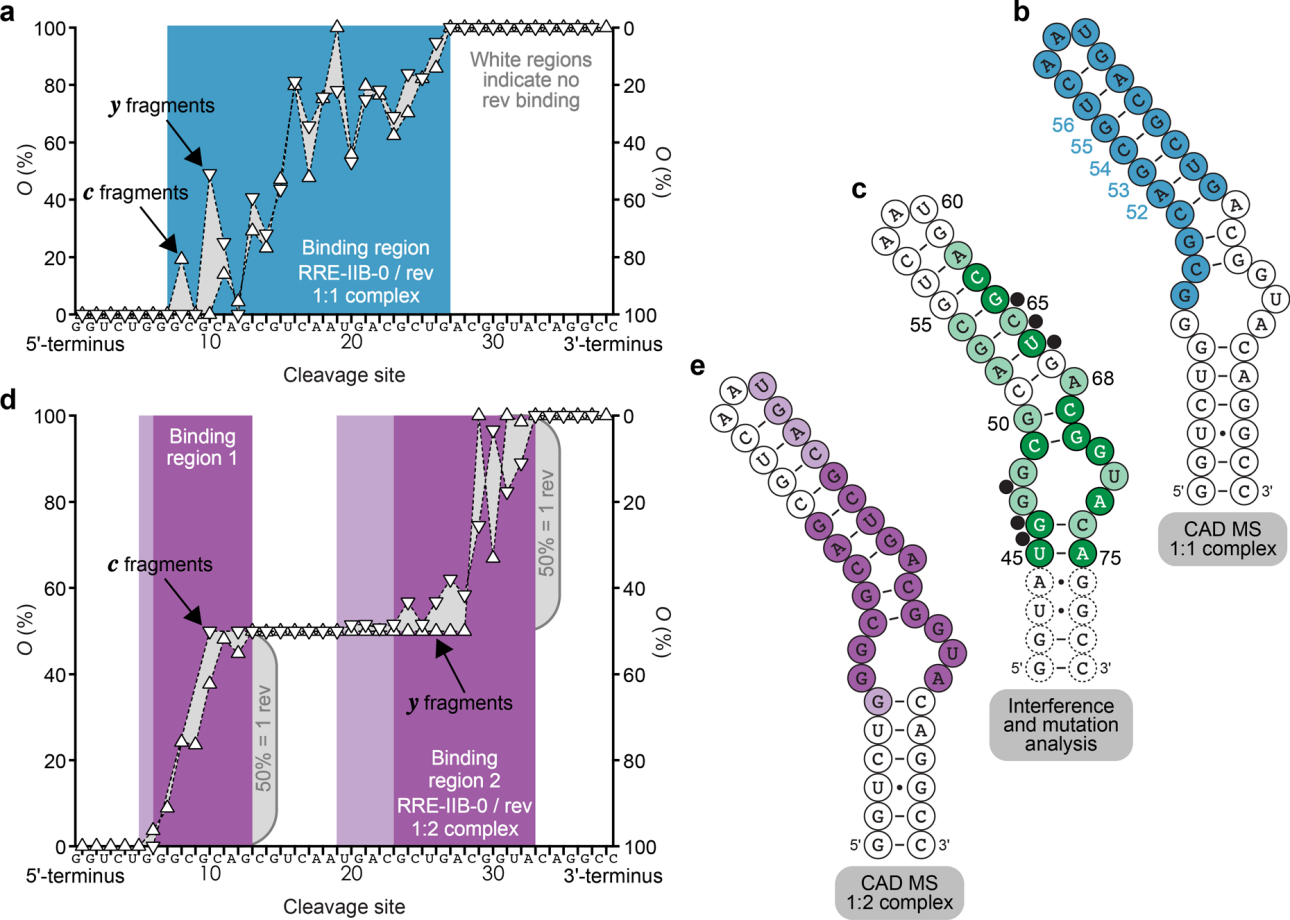
Binding site mapping of RRE-IIB-0/rev complexes by CAD MS. **a** Site-specific occupancies (*O*) of ***c*** (left axis) and ***y*** (right axis) fragments with rev ARM peptide from CAD of 1:1 complex ions, (**RRE-IIB-0** + 1·rev - 15H)^15−^, at 150 eV laboratory frame collision energy and the corresponding binding region (blue) mapped onto the predicted secondary structure of **RRE-IIB-0** (**b**) show poor agreement with binding sites indicated by chemical interference and mutation analysis (**c**). **d** Occupancies of fragments from CAD of 1:2 complex ions, (**RRE-IIB-0** + 2·rev - 16H)^16−^, at 208 eV and corresponding binding sites (violet) mapped onto the **RRE-IIB-0** structure (**e**) show good agreement with chemical interference and mutation analysis data (**c**). Darker and lighter colors in **c**–**e** stand for stronger and weaker binding and interference, respectively, and black circles in **c** illustrate interference with rev binding by phosphate ethylation.

For the **RRE-IIB-0**/rev 1:1 complex, the MS data indicate binding of the rev ARM peptide to G48-G67 (Fig. 3a), which corresponds to the upper stem and loop region of the secondary structure predicted (http://rna.tbi.univie.ac.at)^41^ for **RRE-IIB-0** RNA (Fig. 3b). This binding region strongly disagrees with data from chemical interference and mutagenesis analysis^18,19,26^ and chemical shifts from NMR experiments^42^ that all indicate rev binding to U45-C54 and A62-A75 but no binding to G55-G61 (Fig. 3c). By contrast, our data from CAD MS of the **RRE-IIB-0**/rev 1:2 complex that show binding of the rev ARM peptide to G46-G53 and U60-A73 (Fig. 3d, e) are in excellent agreement with the U45-C54 and A62-A75 binding regions inferred from chemical interference, mutagenesis analysis, and NMR experiments (Fig. 3c). The MS data in Figs. 2, 3 and Supplementary Fig. 4 reveal sequential binding of two rev ARM peptide molecules to **RRE-IIB-0** RNA, with the first peptide binding to the upper stem and loop region. Binding of the second peptide must have caused transfer of the first peptide because in the 1:2 complexes, both peptides bind to the internal loop of RRE stem IIB RNA. In support of this hypothesis, Santos and coworkers found that HIV-1 replication is blocked by a branched peptide that binds to both the upper stem and the internal loop of RRE IIB^43^.

NMR or X-ray structures of RRE IIB RNA with a wild-type upper stem (C51-G67, Fig. 3b, c, e) are not yet available, but structures of different upper-stem-truncated RNA constructs with 58-68% sequence similarity to the 45–75 region of wild-type RRE RNA (PDB entries 1CSL, 1DUQ, 1G70, and 1EBR) and 1:1 (1ETF) and 1:2 (4PMI) complexes with rev ARM peptides have been published (RNAs **RRE-TR-0**, **RRE-TR-2**, **RRE-TR-3**, **RRE-TR-4**, **RRE-TR-5**, Fig. 1). Truncated RNA constructs were designed under the assumption that the C54•G64, G55•C63, and U56•A62 base pairs in the upper stem of RRE IIB RNA and the nucleobases in the loop region are not important for specific binding of rev and rev ARM peptides^44^. For example, it was reported that replacement of the entire upper stem with the stable hairpin-loop sequence CUUCGG did not significantly affect the dissociation constant of RRE/rev complexes^18^. We found here that ESI MS of solutions with **RRE-TR-0** RNA with a truncated upper stem (Fig. 1) and rev ARM peptide produced both 1:1 and 1:2 complexes, similar to **RRE-IIB-0** RNA (Fig. 2a) and consistent with isothermal titration calorimetry (ITC) data from titration of **RRE-TR-0** into ARM rev peptide solutions that were distinctly biphasic^31^ and indicative of two different binding sites of differing binding affinity^45^. The kinetics of rev ARM peptide binding to **RRE-TR-0**, showing first an increase and then a decrease in 1:2 complex yield (see Supplementary Fig. 5), was qualitatively similar to that for **RRE-IIB-0** (Fig. 2b), although binding to **RRE-TR-0** was generally faster. Moreover, the yield of 1:2 complexes at incubation times in the plateau regions was higher for **RRE-TR-0** (~60% at ~0.5–2 h, see Supplementary Fig. 5a) than for **RRE-IIB-0** (~40% at ~1–8 h, Fig. 2b), which suggests that transfer of rev during formation of the 1:2 complex is energetically more demanding for **RRE-IIB-0** than for **RRE-TR-0**.

**Fig. 4:**
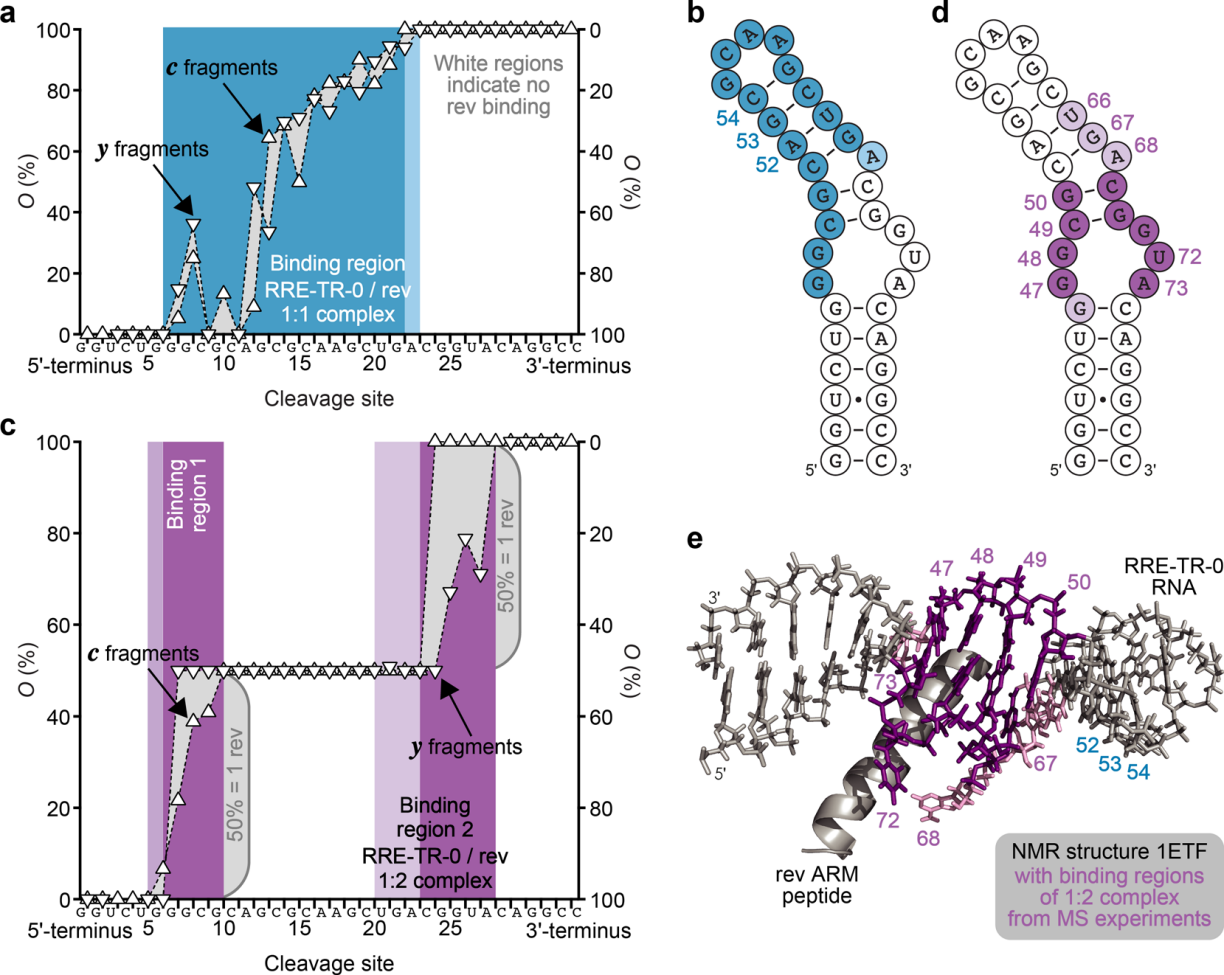
Binding site mapping of RRE-TR-0/rev complexes by CAD MS. **a** Site-specific occupancies (*O*) of ***c*** (left axis) and ***y*** (right axis) fragments with rev ARM peptide from CAD of 1:1 complex ions, (**RRE-TR-0** + 1·rev - 14H)^14−^, at 137.2 eV and the corresponding binding region (blue) mapped onto the predicted secondary structure of **RRE-TR-0** (**b**) show poor agreement with binding sites in the NMR structure (**e**). **c** Occupancies of fragments from CAD of 1:2 complex ions, (**RRE-TR-0** + 2·rev - 14H)^14−^, at 175.5 eV and corresponding binding sites (violet) mapped onto the **RRE-TR-0** structure (**d**) show good agreement with binding sites in the NMR structure (**e**). Darker and lighter colors in **b**, **d**, and **e** stand for stronger and weaker binding, respectively.

For binding site mapping in the 1:1 and 1:2 complexes by CAD, we isolated (**RRE-TR-0** + 1·rev - 14H)^14−^ ions at *m*/*z* ~1000 and (**RRE-TR-0** + 2·rev - 14H)^14−^ ions at *m*/*z* ~1214 (both ~0.4 charges/nt), respectively. CAD of the 1:1 complex produced ***c*** and ***y*** fragments with and without peptide attached (see Supplementary Fig. 6a), from which occupancy values were calculated (Fig. 4a) that revealed rev binding to the upper stem of **RRE-TR-0** (Fig. 4b), similar to the **RRE-IIB-0**/rev 1:1 complex (Fig. 3a, b). CAD of the **RRE-TR-0**/rev 1:2 complex revealed rev binding to G46-G50 and U66-A73 (Fig. 4c, d and Supplementary Fig. 6b); these nucleotides were also part of the binding interface in the **RRE-IIB-0**/rev 1:2 complex (Fig. 3d, e). As illustrated in Fig. 4e, the MS data of the 1:2 (Fig. 4d) but not the 1:1 (Fig. 4c) **RRE-TR-0**/rev complex show excellent agreement with the NMR structure of the **RRE-TR-0**/rev 1:1 complex (PDB entry 1ETF)^24^.

The structure 1ETF in Fig. 4e was derived from NMR experiments with **RRE-TR-0** and rev in solutions at pH 5.5^24^ whereas in our MS experiments, we used a pH of 7.5. Because the binding region indicated by the MS data for the **RRE-TR-0**/rev 1:1 complex (Fig. 4b) did not agree with the NMR data, we wondered if it was affected by solution pH. However, the binding region indicated by data from CAD of (**RRE-TR-0** + 1·rev - 13H)^13−^ ions—the abundance of (**RRE-TR-0** + 1·rev - 14H)^14−^ was too low for CAD at pH ~5.5—was virtually the same at pH ~5.5, ~7.5, and ~9.5 (see Supplementary Fig. 7). Moreover, CAD of (**RRE-TR-0** + 1·rev - nH)^n−^ ions with *n* = 11–15 electrosprayed from a solution at pH ~7.5 (see Supplementary Fig. 8) revealed that A52, G53, and C54 are buried deep within the binding interface of the **RRE-TR-0**/rev 1:1 complex (Fig. 4b, e) (i.e., their interactions with the RNA were least affected by proton mobilization during CAD that can convert strong salt bridges, such as arginine–phosphate interactions into weaker ionic hydrogen bonds, and ionic hydrogen bonds into far weaker neutral hydrogen bonds^6,13,46^) whereas they do not bind to rev peptide in the NMR structure (Fig. 4e). The RNA binding regions of the NMR structure 1ETF instead agree with the MS data of the (**RRE-TR-0** + 2·rev - nH)^n−^ complexes at all n studied (Fig. 4d, e and Supplementary Fig. 9).

Because rev binding to RRE stem IIB RNA is sequential and reversible (Fig. 2c), we further wondered if the binding region in the 1:1 complexes was different for different incubation times. The rev ARM peptide binding regions indicated by CAD of (**RRE-TR-0** + 1·rev - 14)^14−^ ions, recorded ~6 min and ~155 min after preparation of a solution with **RRE-TR-0** RNA (2 µM) and rev ARM peptide (4 µM), before and after the plateau region in Supplementary Fig. 5a, were the same as that at ~60 min within the plateau region (see Supplementary Fig. 10). Because the binding site of **RRE-TR-0**/rev 1:1 complexes did not differ for different incubation times and was very similar to that of the **RRE-IIB-0**/rev 1:1 complexes (Fig. 4a, b), we conclude that it does not result from unspecific binding but instead is the binding site of RRE at which assembly of the functional RRE/rev ribonucleoprotein complex is initiated^22^.

### MS of full-length RRE stem II/rev complexes

**Fig. 5:**
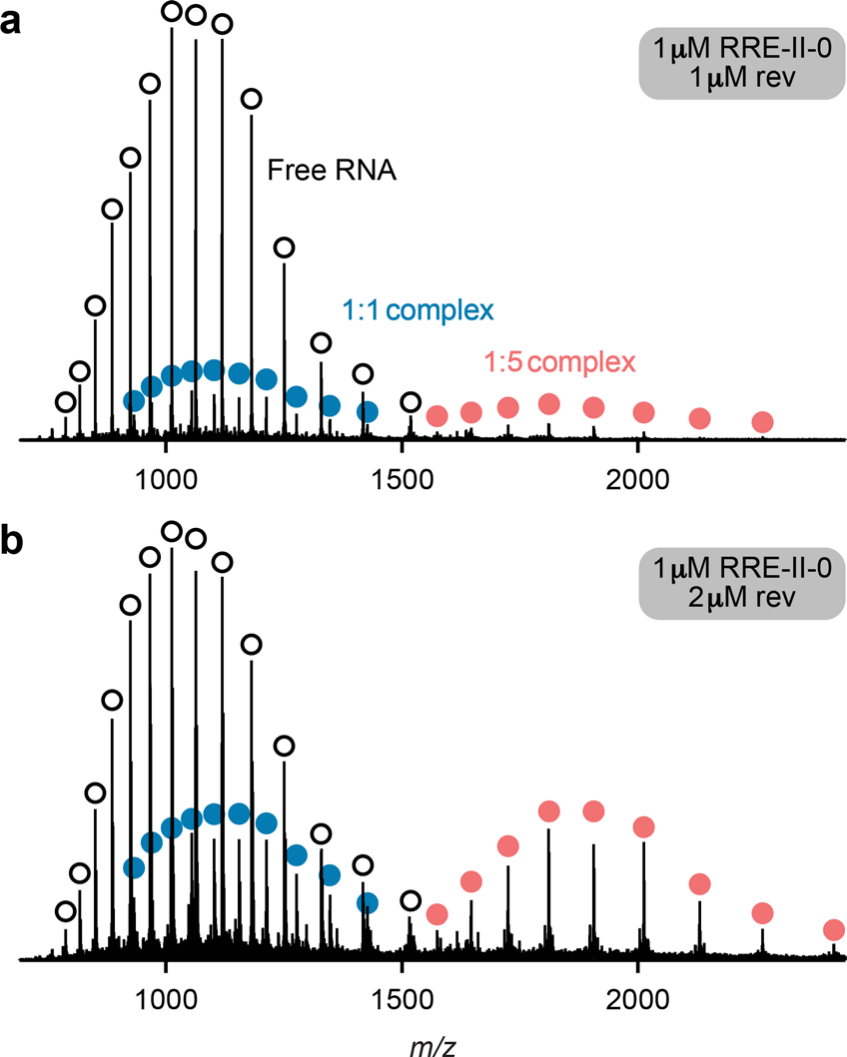
RRE-II-0/rev complex stoichiometry probed by native MS. ESI spectra of 1 µM **RRE-II-0** RNA solutions in 9:1 H_2_O/CH_3_OH with ~1 mM piperidine (pH ~8.1) and **a** 1 µM and **b** 2 µM rev peptide (~10 min incubation time) show signals of free RNA (open circles), 1:1 complex (blue circles), and 1:5 complex (red circles).

**Fig. 6:**
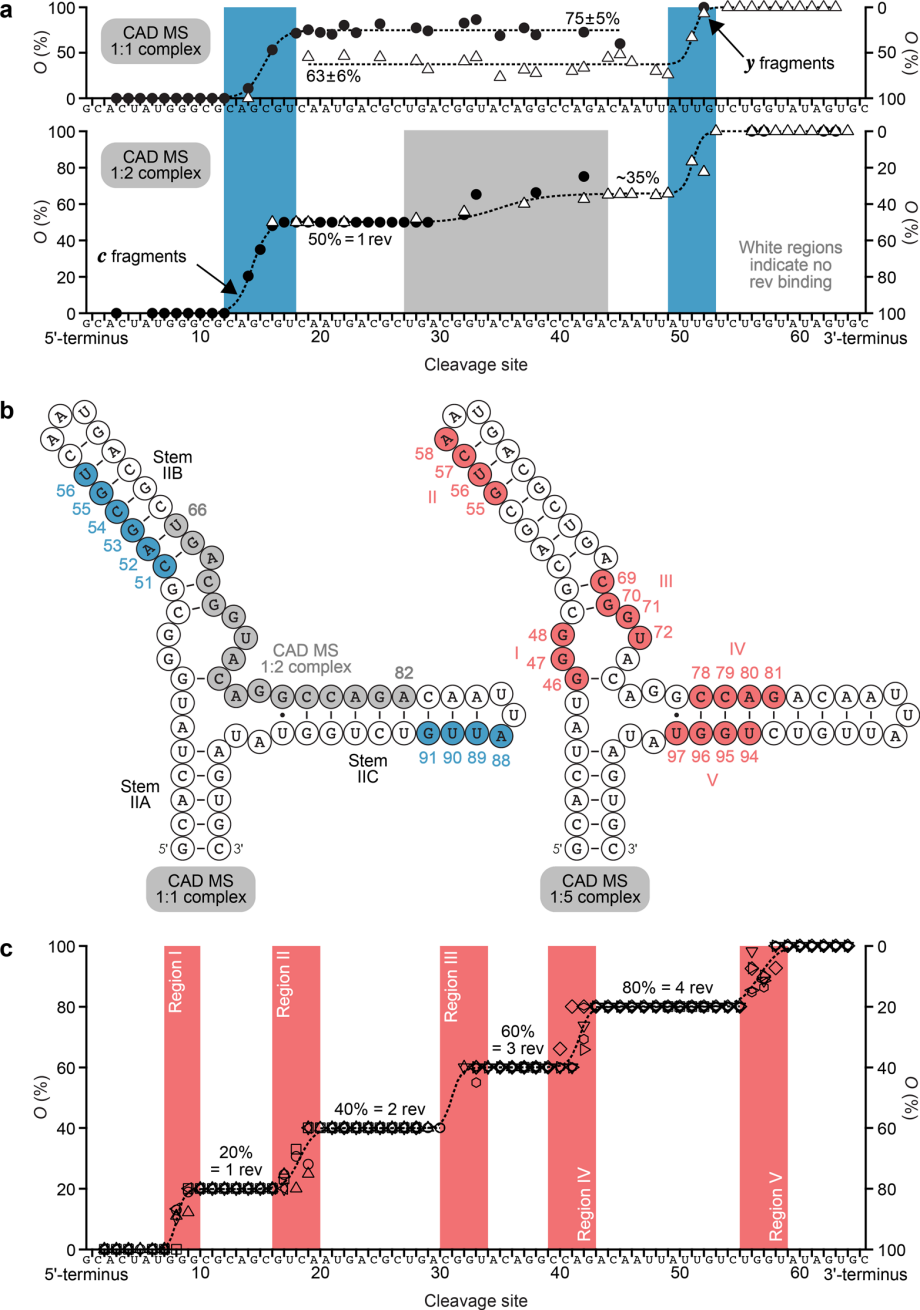
Binding site mapping of RRE-II-0/rev complexes by CAD MS. **a** Site-specific occupancies (*O*) of ***c*** (circles, left axis) and ***y*** (triangles, right axis) fragments with rev ARM peptide from CAD of 1:1 and 1:2 complex ions, (**RRE-II-0** + 1·rev - 20H)^20−^ and (**RRE-II-0** + 2·rev - 20H)^21−^, at 200 eV and 241.5 eV, respectively, and **b** the corresponding binding regions (blue and gray) mapped onto the predicted secondary structure of **RRE-II-0** (with additional hydrogen bonds between G46 and C74, and G77 and U97)^27^; **c** occupancies of fragments from CAD of 1:5 complex ions, (**RRE-II-0** + 5·rev - nH)^n−^, for *n* = 18 at 387 eV (***c***: vertical lozenges, ***y***: horizontal lozenges), *n* = 19 at 399 eV (***c***: leftward triangles, ***y***: rightward triangles), *n* = 20 at 400 eV (***c***: circles, ***y***: hexagons), *n* = 21 at 399 eV (***c***: squares, ***y***: diamonds), *n* = 22 at 418 eV (***c***: upward triangles, ***y***: downward triangles) for which source data are provided as a Source Data file, and the corresponding binding regions (red) mapped onto the **RRE-II-0** structure (**b**).

To further test the hypothesis that the first rev molecule binds to the upper stem of RRE IIB with high specificity, and to investigate the role of the three-way junction from which stem IIB originates, we studied the 66 nt RNA **RRE-II-0** that encompasses stems A, B, and C. ESI of solutions of **RRE-II-0** RNA (1 µM) and rev ARM peptide (1 and 2 µM) at pH ~8.1 (~10 min incubation time) showed free RNA and 1:1 and 1:5 RNA/peptide complexes (Fig. 5). Signals of complexes of intermediate stoichiometry were minor species at the incubation times studied (Fig. 5 and Supplementary Fig. 11), which suggests that they have a far lower stability than the 1:1 and 1:5 complexes and that binding is specific. CAD of the 1:1 complexes revealed rev peptide binding to two regions, C51-U56 and U87-G91, and the higher occupancy value of ***c*** (~75%) compared to that of ***y*** (~63%) fragments in the region 18–49 (see Supplementary Fig. 12) indicates a higher affinity for rev peptide of C51-U56 over U87-G91, consistent with initial rev binding to the upper stem of RRE IIB and additional interactions with stem IIC or rapid exchange of rev between these two sites (Fig. 6a, b). The data for 1:1 complexes of **RRE-TR-0** (Fig. 4a, b), **RRE-IIB-0** (Fig. 3a, b), and **RRE-II-0** (Fig. 6a, b) all show rev ARM peptide binding to C51-U56, of which A52-C54 are buried deep within the binding interface (see Supplementary Fig. 8). Additional interactions with G47-G50 and G48-G50 were observed for **RRE-TR-0** and **RRE-IIB-0**, respectively, but not for **RRE-II-0**, which instead showed binding to A88-G91. This finding suggests that in the 1:1 complexes, rev ARM peptide interacts with G47-G50 (**RRE-TR-0**) or G48-G50 (**RRE-IIB-0**) to compensate for the unavailability of A88-G91 in the stem IIB constructs. CAD of the 1:2 complexes of **RRE-II-0** (from ESI of 1 µM **RRE-II-0** RNA and 3 µM rev ARM peptide solutions, see Supplementary Fig. 11c) showed binding to the exact same sites in ~85% of the complexes, with one peptide binding to C51-U56 and the other to U87-G91. In the other ~15% of the 1:2 complexes, one peptide again binds to C51-U56, and the other peptide to the region U66-A82 (Fig. 6a, b). These data confirm that the first rev molecule binds to the upper stem of RRE IIB with high specificity, and suggest that additional interactions of rev with other sites are important for relay of rev during the assembly of the functional RRE/rev ribonucleoprotein complex.

CAD of the 1:5 complexes of **RRE-II-0** with rev ARM peptide (which were not observed in spectra from ESI of denaturing solutions at pH ~10, see Supplementary Fig. 13) showed binding of one rev peptide molecule to each of the regions G46-G48 (I), G55-A58, (II), C69-U72 (III), C78-G81 (IV), and U94-U97 (V) (Fig. 6c). Regions I and III, and IV and V, are in close proximity to each other in the predicted secondary structure of **RRE-II-0** RNA (Fig. 6b), which would favor rev dimer formation in functional RRE/rev complexes. Moreover, regions III and IV are located within the region U66-A82 that showed rev peptide binding in ~15% of the 1:2 complexes, and region II overlaps with C51-U56, the initial binding region of rev ARM peptide in the 1:1 and 1:2 complexes of **RRE-II-0** RNA. These data strongly suggest a relay mechanism in which the upper stem of RRE IIB (Figs. 3b, 4b, and 6b) continuously recruits and redistributes rev to a total of four sites in RRE stem II that provide a rationale for rev protein dimerization beyond protein–protein interactions^4,47^. In this picture, immediate recognition of the mutation-intolerant, helical ARM region of rev protein^48^ by the conformationally inflexible upper stem of RRE IIB^27,49^ with its highly conserved sequence^25,50^ could account for fast (Fig. 2b and Supplementary Figs. 2, 5) and specific (Figs. 3b, 4b, and 6b) binding of rev. Specific recognition of rev by the upper stem of RRE IIB is further indicated by bacterial reporter system and gel shift assay studies that showed no or very little antitermination activity, and substantially reduced binding of rev ARM peptide to RRE IIB with nucleotide substitutions in the upper stem (C51•G67, A52•U66, G53•C65, C54•G64)^51^. The relay of rev to other binding sites (Figs. 3e, 4d, and 6b) requires structural changes of the overall flexible RRE RNA^52^ (including the internal loop region of stem IIB) that can explain the observed slower binding of additional rev molecules (Fig. 2b and Supplementary Figs. 2, 3, 5). In support of such structural changes, Al-Hashimi and coworkers found that both RRE IIB and RRE II robustly exist in dynamic equilibrium with non-native conformations that have a combined population of ∼20%. Furthermore, for efficient relay of rev, the affinity of the binding site in the upper stem of free RRE IIB should be smaller than the affinities of the binding sites in the final structure. In support of this hypothesis, ITC data indicated two different sites for rev ARM peptide binding to **RRE-TR-0**, one of relatively high (*K*_D_ = 0.027 ± 0.007 nM) and the other of moderate (*K*_D_ = 0.70 ± 0.29 μM) affinity^31^. Although the weaker binding event in the ITC study was interpreted as nonspecific binding^31^, our MS study shows that this binding event is specific (Fig. 4b) and that the binding site is preserved in **RRE-IIB-0** and **RRE-II-0** (Figs. 3b and 6b). Likewise, ITC data for tat peptide binding to TAR RNA indicated a second binding event that was interpreted as nonspecific binding^53^, and very recently highly sophisticated NMR^54^ and native MS^6^ experiments could show that TAR RNA actually does have two different binding sites. Finally, the fact that the initial binding site of **RRE-IIB-0** is still occupied with rev ARM peptide in the 1:5 complexes suggests that it is responsible for relay of rev ARM peptides to RRE sites that are not part of stem II.

## Discussion

Our ESI and CAD MS data show that stem IIB RNA can specifically bind one or two rev ARM peptides, and that full-length RRE stem II RNA forms stable complexes with one or five rev ARM peptides. Binding site mapping by CAD MS revealed that the first rev molecule initially binds to the upper IIB stem in all RNA constructs studied here. The different binding sites in the 1:1 and 1:2 complexes of stem IIB RNA suggest that binding of the second rev ARM peptide causes transfer of the first rev ARM peptide, such that both peptides bind to the well-characterized regions that include the internal loop. Binding of the first rev ARM peptide to the upper IIB stem of full-length RRE II RNA, followed by facile binding of four additional rev ARM peptides, further supports our proposed relay mechanism. In this picture, the first rev molecule binds to the upper IIB stem, from where it is relayed—aided by additional interactions with A88-G91—to regions III or IV such that the next rev molecule can again bind to the upper IIB stem. Sequential binding to the upper IIB stem and relay of a total of four rev molecules to regions I, III, IV, and V allows for dimer formation between the rev molecules bound to regions I and III, and those bound to regions IV and V. Moreover, our data from ESI experiments with isotopically labeled rev show that sequential binding of rev to RRE RNA is a highly dynamic process in which rev exchanges at a rate that exceeds the observed rate of complex formation. The label-free, native CAD MS approach introduced here for RNA of up to 66 nt requires only small amounts of sample, complements more established techniques in integrative structural biology studies of RNA/protein complexes, and suggests the upper stem of RRE IIB as a primary target for the development of drugs against HIV-1 infection.

## Methods

### RNA, peptides, and chemicals

RNA was prepared by solid phase synthesis^55,56^, purified by HPLC, and desalted^6^ by diluting ~100 µl RNA solution in H_2_O (~50 µM) with ~400 µl aqueous ammonium acetate (100 mM) solution, followed by concentration to ~100 µl using centrifugal concentrators (Vivaspin 500, MWCO 5000, Sartorius AG, Germany); the concentration-dilution process was repeated five times and followed by six cycles of concentration and dilution with H_2_O. **RRE-TR-0** RNA (5′-GGUCU GGGCG CAGCG CAAGC UGACG GUACA GGCC-3′, calculated and measured monoisotopic mass *m*_c_ = 11,017.538 Da and *m*_m_ = 11,017.534 Da, respectively), **RRE-IIB-0** RNA (5′-GGUCU GGGCG CAGCG UCAAU GACGC UGACG GUACA GGCC-3′, *m*_c_ = 12,608.729 Da; *m*_m_ = 12,608.731 Da), and **RRE-II-0** RNA (5′-GCACU AUGGG CGCAG CGUCA AUGAC GCUGA CGGUA CAGGC CAGAC AAUUA UUGUC UGGUA UAGUG C-3′, *m*_c_ = 21,269.83 Da; *m*_m_ = 21,269.79 Da) sequences were confirmed by CAD MS^40^. The rev ARM peptide (23 residues, H_2_N-DTRQA RRNRR RRWRE RQRAA AAR-OH^24^) trifluoroacetate salts were custom synthesized by ThermoFisher Scientific, USA (unlabeled peptide, >98%, *m*_c_ = 2991.6649 Da and *m*_m_ = 2991.6645 Da) and Sigma-Aldrich, Austria (isotopically labeled peptide with ^13^C and ^15^N at R3, R6, and R7, >96%, *m*_c_ = 3021.6898 Da and *m*_m_ = 3021.6893 Da). Peptides were desalted by diluting ~200 µl rev peptide solution in H_2_O (~1 mM) with 1.8–3.8 ml aqueous ammonium acetate (100 mM) solution, followed by concentration to 200–500 µl using centrifugal concentrators (Microsep Advance, MWCO 1000, Pall Corporation, USA, or Vivaspin 2, MWCO 2000, Sartorius AG, Germany); the concentration-dilution process was repeated 4–5 times and followed by 5–6 cycles of concentration and dilution with H_2_O. The rev ARM peptide sequence was confirmed by electron capture dissociation (ECD) MS^57^. H_2_O was purified to 18 MΩ cm at room temperature using a Milli-Q system (Millipore, Austria), CH_3_OH (Acros, Austria) was HPLC-grade, ammonium acetate (≥99.0%) and piperidine (≥99.5%) were from Sigma-Aldrich (Austria).

### Sample preparation and electrospray ionization

Prior to the addition of rev peptide solution, RNA solutions were heated to 90 °C for 90 s, followed by cooling on ice for ~10 min and thermal equilibration for another ~10 min at room temperature. Solutions of RNA (1–2 µM) and peptide (0.5–5 µM) were electrosprayed at a flow rate of 1.5 µl min^−1^ in negative ion mode; the potential difference between the ESI emitter (nebulizer) and the inlet capillary was 3100 or 3200 V and the desolvation gas (N_2_) temperature 180 °C. For native ESI, 1–1.25 mM piperidine^58^ was added to RNA/peptide solutions in 9:1 H_2_O/CH_3_OH (pH ~7.5 to ~8.1); our previous study on TAR/tat^6^ showed that the addition of 10% CH_3_OH at near-neutral pH did not alter the RNA/peptide binding region established by NMR spectroscopy. Further, data from chemical probing experiments with *N*-cyclohexyl-*N*′-(2-morpholinoethyl)carbodiimide methyl-*p*-toluenesulfonate (CMCT) were, within error limits, the same for solutions with and without 10% CH_3_OH at pH ~8 (see Supplementary Fig. 1). For ESI under denaturing conditions, piperidine was added to RNA/peptide solutions in 1:1 H_2_O/CH_3_OH at a concentration of 25 mM (pH ~10).

### FT-ICR mass spectrometry

All experiments were performed on a 7 T FT-ICR instrument (Apex Ultra, Bruker, Austria) equipped with an ESI source, a linear quadrupole for ion isolation, a collision cell for CAD^59^ (Argon gas flow through the collision cell was 0.2 l s^−1^), and an indirectly heated hollow cathode for electron capture dissociation (ECD)^57^. Electrostatic potentials and radiofrequency voltages of ion transfer elements (funnel, hexapole, quadrupole, and ion lenses) were optimized for maximum ion transmission, and the skimmer potential^58^ adjusted for efficient ion desolvation and dissociation of piperidine adducts (40 V for **RRE-TR-0** and **RRE-IIB-0**, and 60 V for **RRE-II-0**). ESI spectra were obtained by operation of the linear quadrupole in transmission (radiofrequency-only) mode. For CAD, ions of interest were isolated in the quadrupole and dissociated in the collision cell using the laboratory frame collision energy indicated in the Figure legends (calculated as the bias potential difference between the ion funnel and the collision cell’s hexapole times the charge of the ion)^59^. However, as discussed in detail in ref. ^59^, these collision energy values are only a relative measure of the energy available for dissociation. As a more general guideline, the laboratory-frame energy for CAD was adjusted for full sequence coverage and minimum secondary dissociation^6,59^ in each experiment. For ESI and CAD, 25–50 and 250–500 scans were added for each spectrum, respectively, and data reduction utilized the SNAP2 algorithm (Bruker, Austria).

### Data reduction

Yields of free RNA, 1:1 complex, and 1:2 complex were calculated from the corresponding signal heigths in the spectra from ESI in negative ion mode, divided by the number of charges as ICR detector response is proportional to this number^60^. Only signals with a signal-to-noise ratio >3 and a mass accuracy <10 ppm were considered for analysis. Signals of rev ARM peptide monomers were observed in the ESI spectra in negative ion mode, but not included in the analysis as the ionization of compounds with high pI—that of rev ARM peptide is >12—in negative ion mode (wrong-way-round ESI) is far less efficient than in right-way-round ESI^61^. Likewise, the ionization efficiency of RNA in positive ion mode (wrong-way-round) is substantially reduced compared with ESI in negative ion mode (right-way-round)^38^. Occupancy values (*O*, in %)^6^ of RRE RNA fragments with rev ARM peptide were calculated for each RNA cleavage site from site-specific yields (*Y*) of ***c*** and ***y*** fragments from CAD with 100% corresponding to the total number of rev molecules bound in each experiment, for CAD of 1:1 complexes as *O* = 100·*Y*(***c*** or ***y*** with 1·rev)/(*Y*(***c*** or ***y*** with 1·rev) + *Y*(***c*** or ***y*** without rev)). For CAD of 1:2 complexes, occupancy values were calculated as *O* = 50·*Y*(***c*** or ***y*** with 1·rev)/(*Y*(***c*** or ***y*** with 1·rev) + *Y*(***c*** or ***y*** without rev)) when *Y*(***c*** or ***y*** with 2·rev) = 0, and as *O* = 50 + 50·*Y*(***c*** or ***y*** with 2·rev)/(*Y*(***c*** or ***y*** with 2·rev)+ *Y*(***c*** or ***y*** with 1·rev)) when *Y*(***c*** or ***y*** without rev) = 0. For CAD of 1:5 complexes, occupancy values were calculated accordingly such that the addition of each rev ARM peptide corresponds to an increase of 20%, i.e., *O* = 20·*Y*(***c*** or ***y*** with 1·rev)/(*Y*(***c*** or ***y*** with 1·rev) + *Y*(***c*** or ***y*** without rev)) when *Y*(***c*** or ***y*** with ≥2·rev) = 0, as *O* = 20 + 20·*Y*(***c*** or ***y*** with 2·rev)/(*Y*(***c*** or ***y*** with 2·rev)+ *Y*(***c*** or ***y*** with 1·rev)) when *Y*(***c*** or ***y*** with ≥3·rev) = 0, as *O* = 40 + 20·*Y*(***c*** or ***y*** with 3·rev)/(*Y*(***c*** or ***y*** with 3·rev) + *Y*(***c*** or ***y*** with 2·rev)) when *Y*(***c*** or ***y*** with ≥4·rev) = 0, as *O* = 60 + 20·*Y*(***c*** or ***y*** with 4·rev)/(*Y*(***c*** or ***y*** with 4·rev)+ *Y*(***c*** or ***y*** with 3·rev)) when *Y*(***c*** or ***y*** with ≥5·rev) = 0, and as *O* = 80 + 20·*Y*(***c*** or ***y*** with 5·rev)/(*Y*(***c*** or ***y*** with 5·rev)+ *Y*(***c*** or ***y*** with 4·rev)) when *Y*(***c*** or ***y*** with 5·rev) ≠ 0. Data from our previous study on TAR/tat^6^ showed that standard deviations of occupancy values varied between 0.1% and 7.9%, with average and median values of ~1.9% and 1.3%, respectively.

### Error estimation for yield data

Errors for the yield data (Fig. 2b and Supplementary Figs. 2, 5) were estimated from TAR-tat binding data obtained in off-line ESI experiments under the same experimental conditions (data from ref. ^6^, also listed in the Source Data file) by calculation of the standard deviation of residuals from exponential fit functions as 3*σ* = 6%. Errors in yield ratio (Fig. 2c and Supplementary Figs. 2, 5b, 5d) were conservatively estimated by addition of the relative errors in yield.

### Reporting summary

Further information on research design is available in the Nature Research Reporting Summary linked to this article.

## Supplementary information

Supplementary Information

Reporting Summary

## Data Availability

All relevant data are available from the corresponding author on reasonable request. Source data are provided with this paper.

## Source data

Source Data
