# Supplementary material for: Native mass spectrometry reveals the initial binding events of HIV-1 rev to RRE stem II RNA
Source: Nat Commun. 2020 Nov 13;11:5750. doi: 10.1038/s41467-020-19144-7 (PMC7666190; doi:10.1038/s41467-020-19144-7)
Supplement: Supplementary file 1 — Supplementary Information [file 41467_2020_19144_MOESM1_ESM.pdf]

Native mass spectrometry reveals the initial binding events of HIV-1 rev to RRE stem II RNA

Eva-Maria Schneeberger,[#,1] Matthias Halper,[#] Michael Palasser, Sarah Viola Heel, Jovana Vušurović, Raphael Plangger, Michael Juen,[2] Christoph Kreutz, Kathrin Breuker*

Institute of Organic Chemistry and Center for Molecular Biosciences Innsbruck (CMBI), University of Innsbruck, Innrain 80/82, 6020 Innsbruck, Austria

* To whom correspondence should be addressed. Tel: +43 512 507 57740; Fax: +43 512 507 57799; email: kathrin.breuker@uibk.ac.at, webpage: https://www.uibk.ac.at/organic/breuker/

[#] these authors contributed equally

[1] present address: Boston Children's Hospital, Harvard Medical School, Boston, MA 02115, USA

[2] present address: Roche Diagnostics GmbH, 82377 Penzberg, Germany.

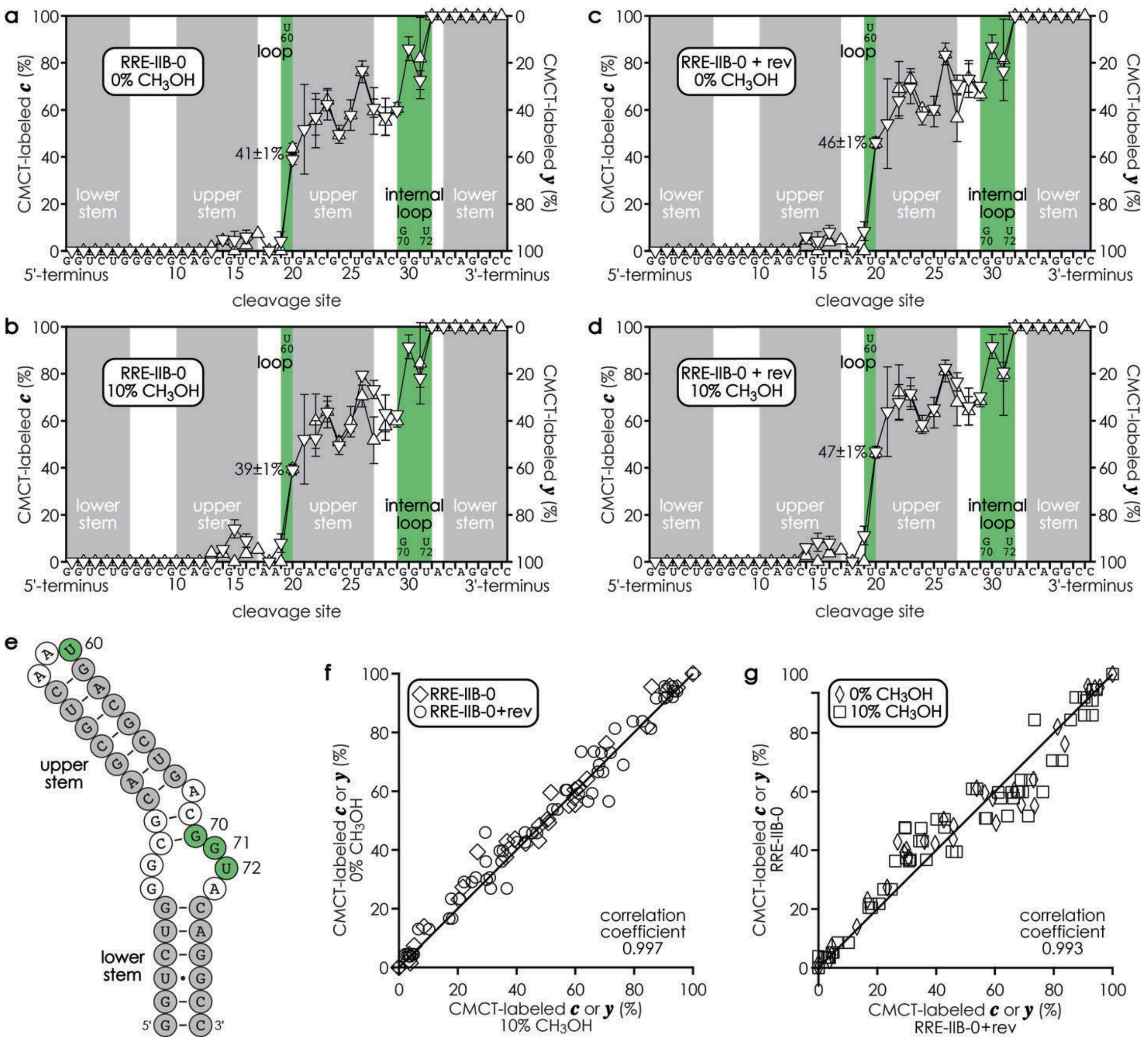

Supplementary Figure 1. Chemical probing experiments showed no effect of 10% CH_3OH on RNA and RNA/rev complex structure. RRE-IIB-0 RNA (30 μM) or **RRE-IIB-0** RNA (30 μM) and rev ARM peptide (60 μM) in buffer solution (12 mM ammonium bicarbonate and 38 mM ammonium acetate, pH 8.0), with or without 10% CH_3OH, were incubated for 1 h, after which *N*-cyclohexyl-*N'*-(2-morpholinoethyl)carbodiimide methyl-*p*-toluenesulfonate (CMCT, 50 mM) was added and allowed to react for 25 min at room temperature (these conditions produced negligible yields,~2%, of multiply labeled RNA to limit possible changes in RNA structure caused by labeling). Reactions were quenched by ~25-fold dilution of the samples and adjustment of the pH to 5 (90 mM ammonium acetate, 11 mM acetic acid). Next, the RNA was desalted and the peptide washed away in consecutive concentration/dilution cycles (1 cycle with 5 M ammonium acetate, followed by heating to 90ºC for 120 s and 2x 5 M ammonium acetate, 1x 2 M ammonium acetate, 3x 100 mM ammonium acetate, 7x H_2O) using centrifugal concentrators (Vivaspin 500, MWCO 5000, Sartorius, Germany). The desalted RNA was electrosprayed from 1.5 μM solutions in 1:1 H_2O/CH_3OH with 2 mM piperidine and 6 mM imidazole, the RNA ions with one CMCT label and a net charge of 9- at m/z ~1428 isolated and dissociated by CAD. The site-specific percentage of CMCT-labeled c and y fragments (shown in a-d as upward and downward triangles, respectively) was calculated from relative abundances of c and y fragments with and without label. The modification patterns in a-d were highly similar for all four solutions, i.e., a) **RRE-IIB-0** without CH_3OH; b) **RRE-IIB-0** with 10% CH_3OH; c) **RRE-IIB-0** and rev without CH_3OH; d) **RRE-IIB-0** and rev with 10% CH_3OH (this experiment was performed twice for error calculation; shown in d are average values), and showed no or very little reaction of CMCT with nucleobases in the lower and upper stem regions (e), from which we conclude that the secondary structure is neither affected by rev binding nor by the addition of 10% CH_3OH. The most reactive nucleobases (`U60`, `G70`, `U72`) are located in the loop and internal loop regions (a-e), and the reactivity of `U60` was not affected by the addition of 10% CH_3OH (41±1% for 0% CH_3OH and 39±1% for 10% CH_3OH), but increased by ~6% upon rev ARM peptide binding (46±1% for 0% CH_3OH and 47±1% for 10% CH_3OH). Further, the percentage values of CMCT-labeled c and y fragments of **RRE-IIB-0** in 0% and 10% CH_3OH solutions showed a higher correlation (f) than the percentage values of CMCT-labeled c and y fragments of **RRE-IIB-0** in the presence and absence of rev ARM peptide (g). This finding could indicate changes in tertiary RNA structure upon rev ARM peptide binding, but a more detailed interpretation of the data in a-d is complicated by the presence of free RNA and both 1:1 and 1:2 RNA/peptide complexes in solutions with a 1:2 stoichiometry of **RRE-IIB-0** RNA and rev ARM peptide (Figure 2b). Error bars in a-d represent ±2·σ from the duplicate measurements (n=2); source data are provided as a Source Data file.

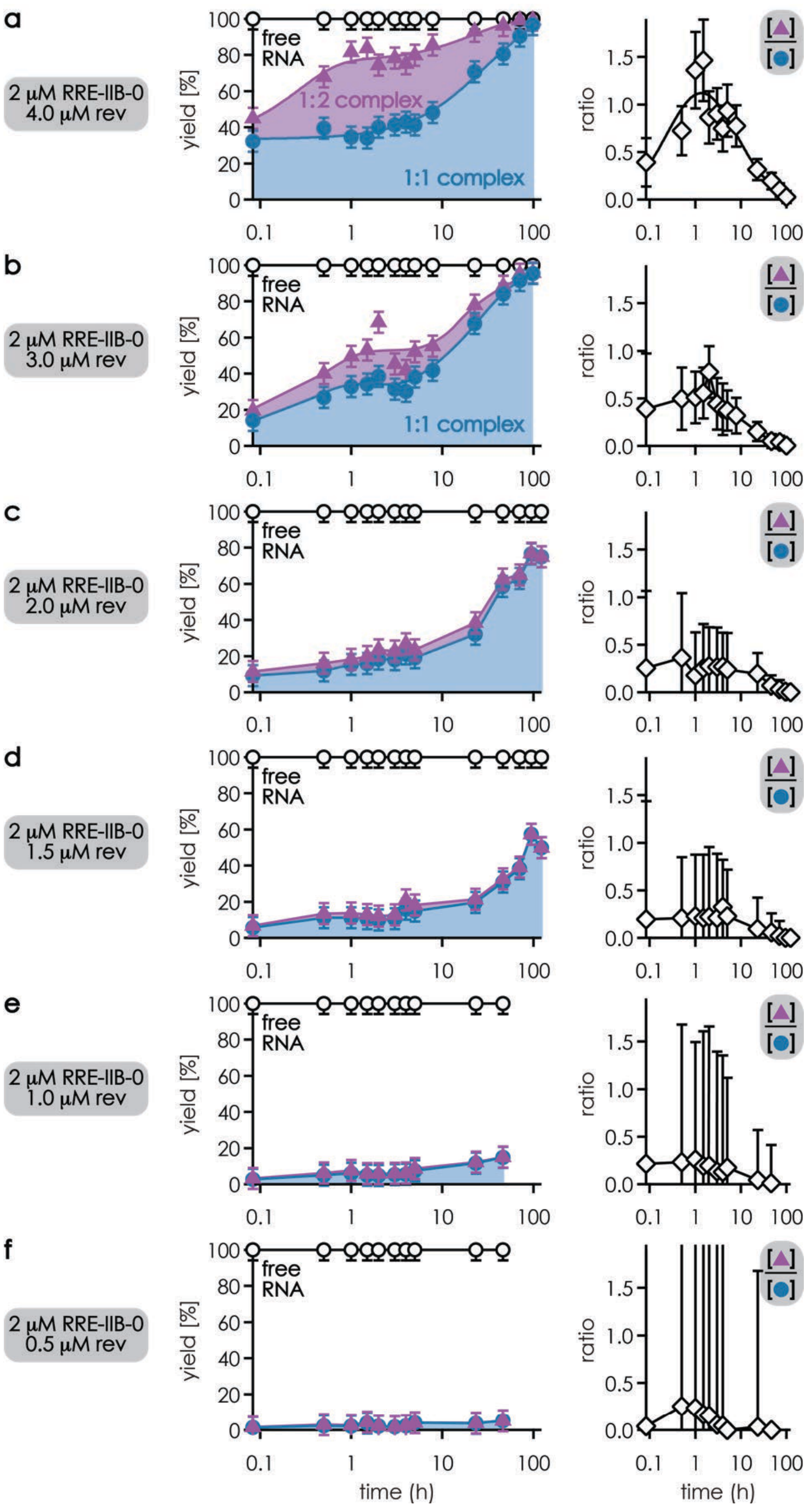

Supplementary Figure 2. Kinetics of RRE-IIB-0/rev association probed by native ESI MS. Stacked area plots (left) illustrating the fractions of free RNA (open circles), 1:1 complex (filled circles), and 1:2 complex (triangles) from ESI of **RRE-IIB-0** RNA (2 μM) and a) 4 μM, b) 3 μM, c) 2 μM, d) 1.5 μM, e) 1 μM, f) 0.5 μM rev peptide solutions in 9:1 H_2O/CH_3OH (pH ~7.5, adjusted by addition of ~1.25 mM piperidine) and corresponding ratio of 1:2 to 1:1 complex yields (right) versus incubation time. Errors in yield were calculated as described in the Methods section, and source data are provided as a Source Data file.

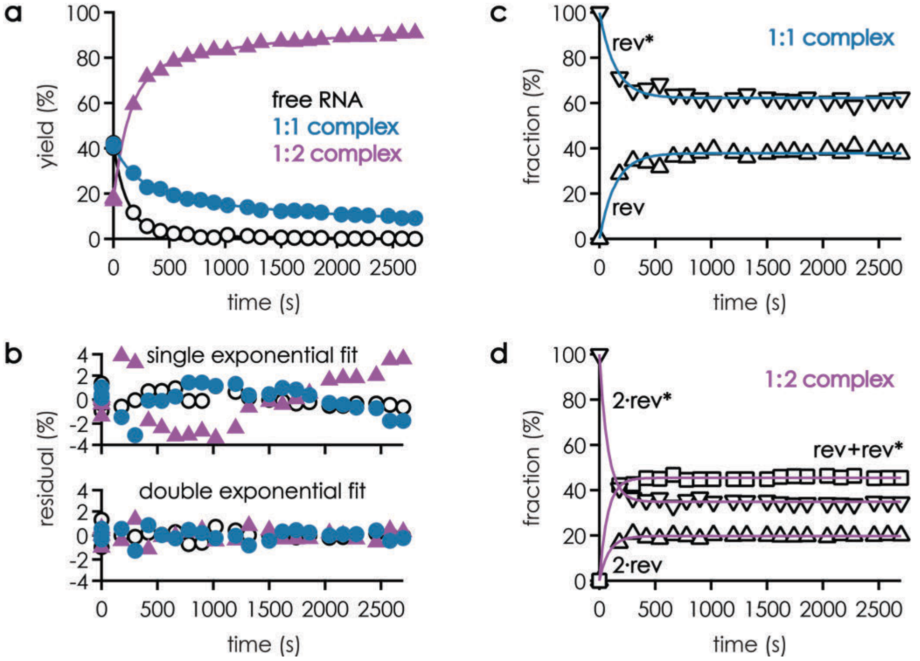

Supplementary Figure 3. Dynamics of rev association and dissociation probed by native ESI. Complex formation and exchange of rev ARM peptide monitored by online-ESI after addition of rev peptide to a solution of **RRE-IIB-0** RNA (2 μM) and isotopically labeled rev peptide (rev*, 4 μM) in 9:1 H_2O/CH_3OH (pH ~7.7, adjusted by addition of ~1.25 mM piperidine) that was prepared 2.5 h before addition of rev (2 μM). a) Yields of free RNA (open circles), 1:1 complex (filled circles), and 1:2 complex (triangles) with double exponential fit functions and b) residuals from nonlinear least-squares fitting with single and double exponential functions; c) fractions of rev (upward triangles) and rev* (downward triangles) in 1:1 complexes and d) fractions of 2·rev (upward triangles), rev+rev* (squares), and 2·rev* (downward triangles) in 1:2 complexes with single exponential fit functions.

		rate v_{slow} [s^{-1}] (preexponential factor [%])	rate v_{fast} [s^{-1}] (preexponential factor [%])	rate v_{faster} [s^{-1}]
yield	free RNA	0.0008 ± 0.0010 (3.1 ± 0.9)	0.0079 ± 0.0006 (38.3 ± 1.6)	-
	1:1 complex	0.0007 ± 0.0003 (16.4 ± 1.2)	0.0050 ± 0.0008 (18.2 ± 2.7)	-
	1:2 complex	0.0008 ± 0.0002 (-20.1 ± 1.1)	0.0069 ± 0.0004 (-55.3 ± 2.2)	-
fraction	2·rev in 1:2 complex	-	-	0.012 ± 0.001
	rev+rev* in 1:2 complex	-	-	0.014 ± 0.001
	2·rev* in 1:2 complex	-	-	0.013 ± 0.001
	rev in 1:1 complex	-	0.0070 ± 0.0007	
	rev* in 1:1 complex	-	0.0070 ± 0.0007	

Supplementary Table 1. Analysis of observed rates from data in Supplementary Figure 3. Observed rates v from nonlinear least-squares fitting of the data in Supplementary Figure 3 with single ($f(t)=A_0+A\cdot exp(-v\cdot t)$, for fractions) and double ($f(t)=A_0+A_1\cdot exp(-v_1\cdot t)+A_2\cdot exp(-v_2\cdot t)$, for yields) exponential functions, categorized as 'slow' (v_{slow}, formation of 1:2 complexes), 'fast' (v_{fast}, formation of 1:1 complexes), and 'faster' (v_{faster}, exchange of rev ARM peptide).

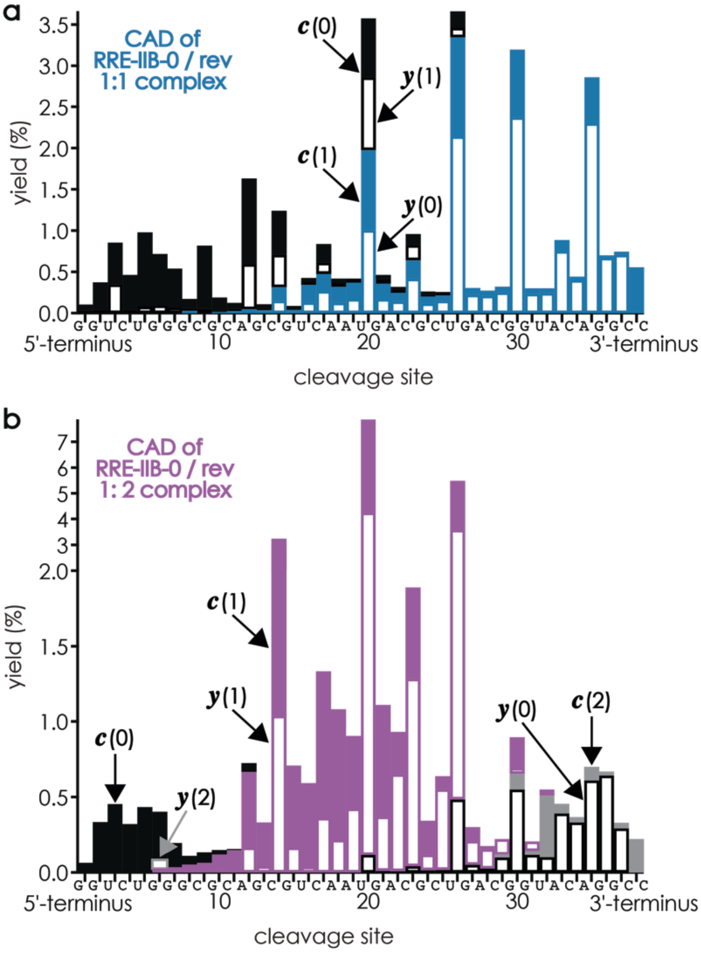

Supplementary Figure 4. Yield of RRE-IIB-0 fragments with and without rev. Site-specific yield of *c* and *y* fragments without (0), with one (1), and with two (2) rev ARM peptides attached from CAD of a) 1:1 complex ions, (**RRE-IIB-0**+1·rev-15H)$^{15-}$, at 150 eV and b) 1:2 complex ions, (**RRE-IIB-0**+2·rev-16H)$^{16-}$, at 208 eV.

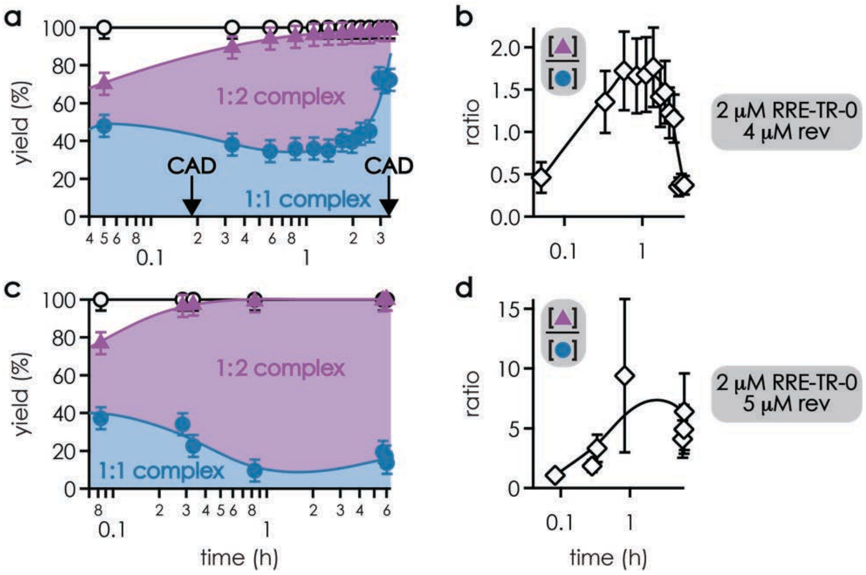

Supplementary Figure 5. RRE-TR-0/rev complex stoichiometry probed by native MS. Stacked area plots illustrating the fractions of free RNA (open circles), 1:1 complex (filled circles), and 1:2 complex (triangles) from ESI of **RRE-TR-0** RNA (2 μM) and a) 4 μM and c) 5 μM rev peptide solutions in 9:1 H_2O/CH_3OH (pH ~7.5, adjusted by addition of ~1.25 mM piperidine) and b), d) the corresponding ratio of 1:2 to 1:1 complex yields (right) versus incubation time. Arrows in a) indicate incubation times at which CAD spectra of 1:1 complexes were recorded (see Supplementary Figure 10). Errors in yield were calculated as described in the Methods section, and source data are provided as a Source Data file.

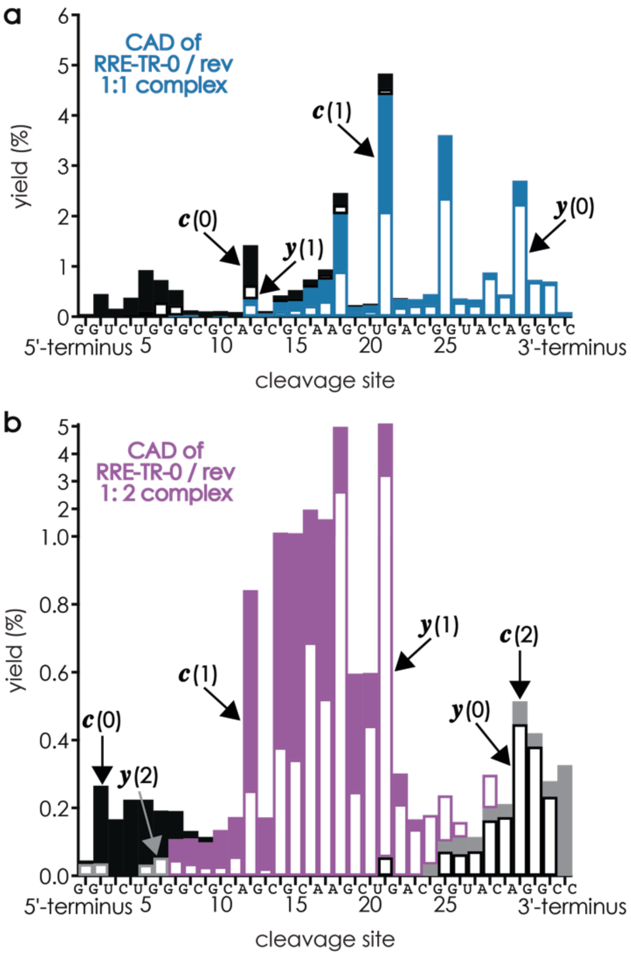

Supplementary Figure 6. Yield of RRE-TR-0 fragments with and without rev. Site-specific yield of c and y fragments without (0), with one (1), and with two (2) rev ARM peptides attached from CAD of a) 1:1 complex ions, (**RRE-TR-0**+1·rev-14H)$^{14-}$, at 137.2 eV and b) 1:2 complex ions, (**RRE-TR-0**+2·rev-14H)$^{14-}$, at 189 eV.

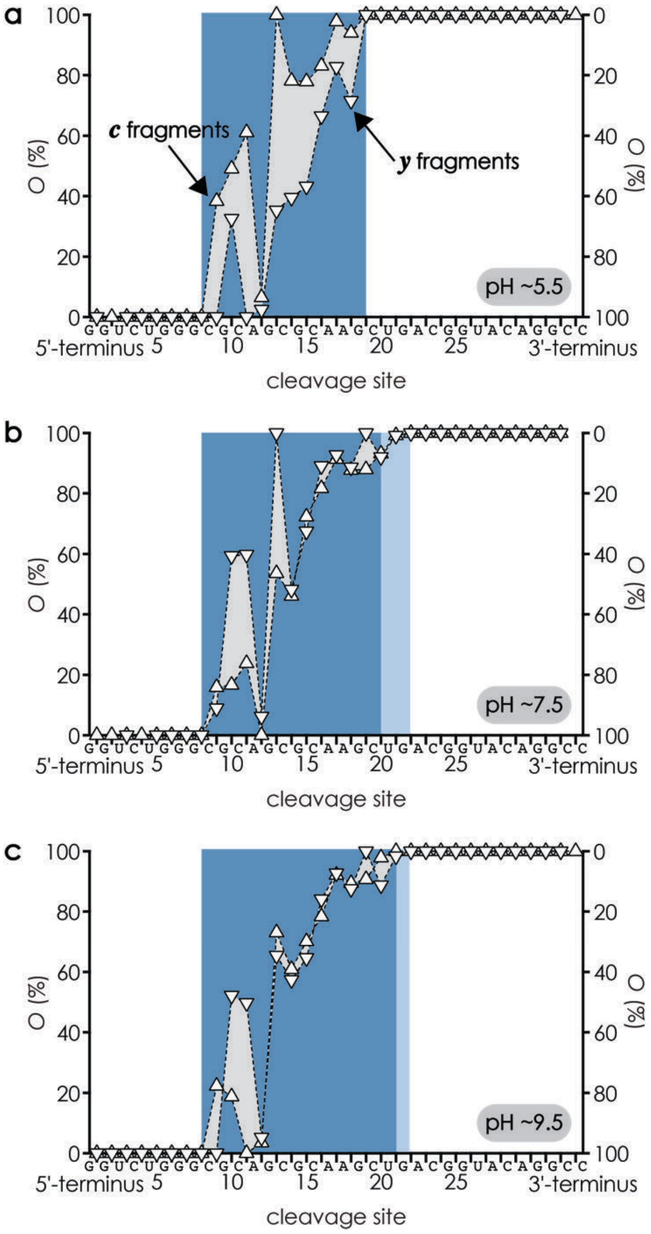

Supplementary Figure 7. Binding site mapping at different pH. Site-specific occupancy (*O*) of *c* (upward triangles, left axis) and *y* (downward triangles, right axis) fragments with rev ARM peptide from CAD (137.8 eV) of 1:1 complex ions, (**RRE-TR-0**+rev-13H)$^{13-}$, electrosprayed from solutions in 9:1 H_2O/CH_3OH at pH a) ~5.5 (~500 μM piperidine), b) ~7.5 (~1.25 mM piperidine), and c) ~9.5 (~3 mM piperidine); regions highlighted in blue indicate non-zero occupancy with weaker binding in light blue.

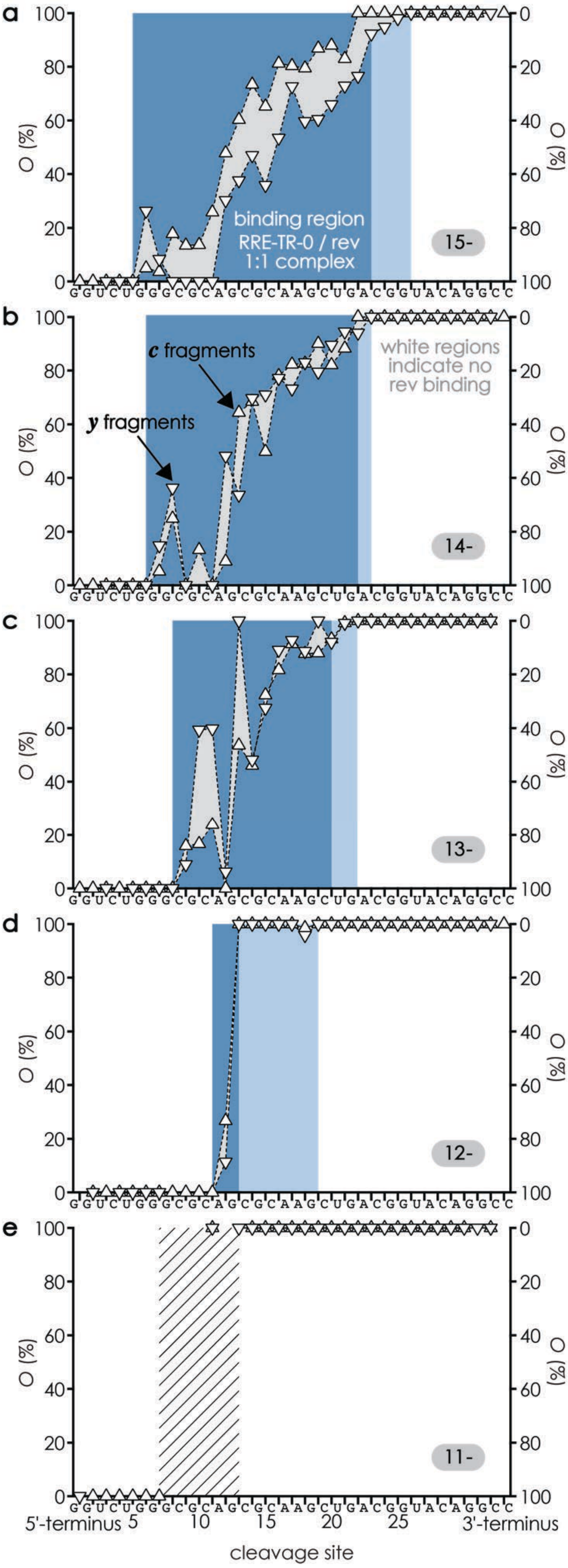

Supplementary Figure 8. Binding site mapping at different 1:1 complex ion net charge. Site-specific occupancy of *c* (upward triangles, left axis) and *y* (downward triangles, right axis) fragments with rev ARM peptide from CAD of 1:1 complex ions, (**RRE-TR-0**+rev-nH)$^{n-}$, electrosprayed from solutions in 9:1 H_2O/CH_3OH at pH ~7.5 (~1.25 mM piperidine) for a) n=15 at 138.0 eV, b) n=14 at 137.2 eV, c) n=13 at 137.8 eV, d) n=12 at 138.0 eV, and e) n=11 at 137.5 eV; regions highlighted in blue indicate non-zero occupancy and the stripes in e) highlight the transition region from 0% to 100%.

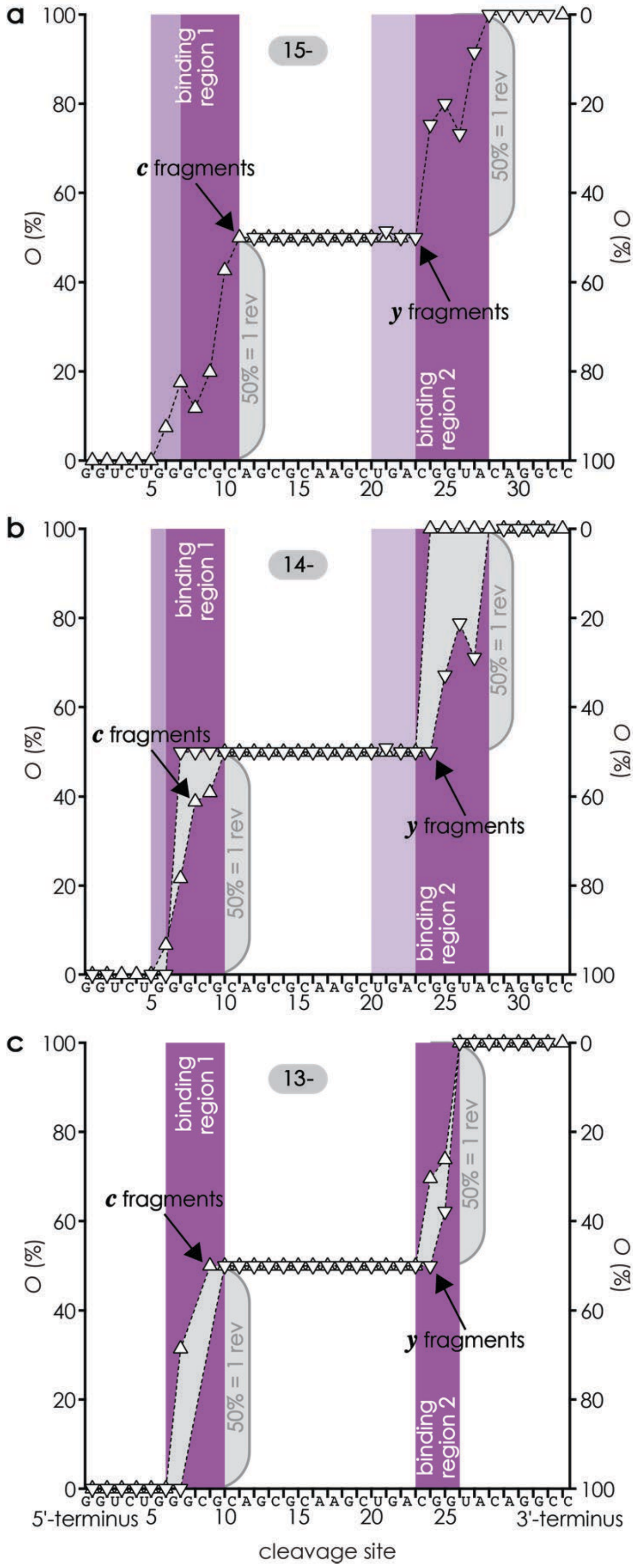

Supplementary Figure 9. Binding site mapping at different 1:2 complex ion net charge. Site-specific occupancy of *c* (upward triangles, left axis) and *y* (downward triangles, right axis) fragments with rev ARM peptide from CAD of 1:2 complex ions, (**RRE-TR-0**+2rev-nH)$^{n-}$, electrosprayed from solutions in 9:1 H_2O/CH_3OH at pH ~7.5 (~1.25 mM piperidine) for a) n = 15 at 187.5 eV, b) n = 14 at 189 eV, c) n = 13 at 188.5 eV; the regions highlighted in purple indicate non-zero occupancy with weaker binding in light purple.

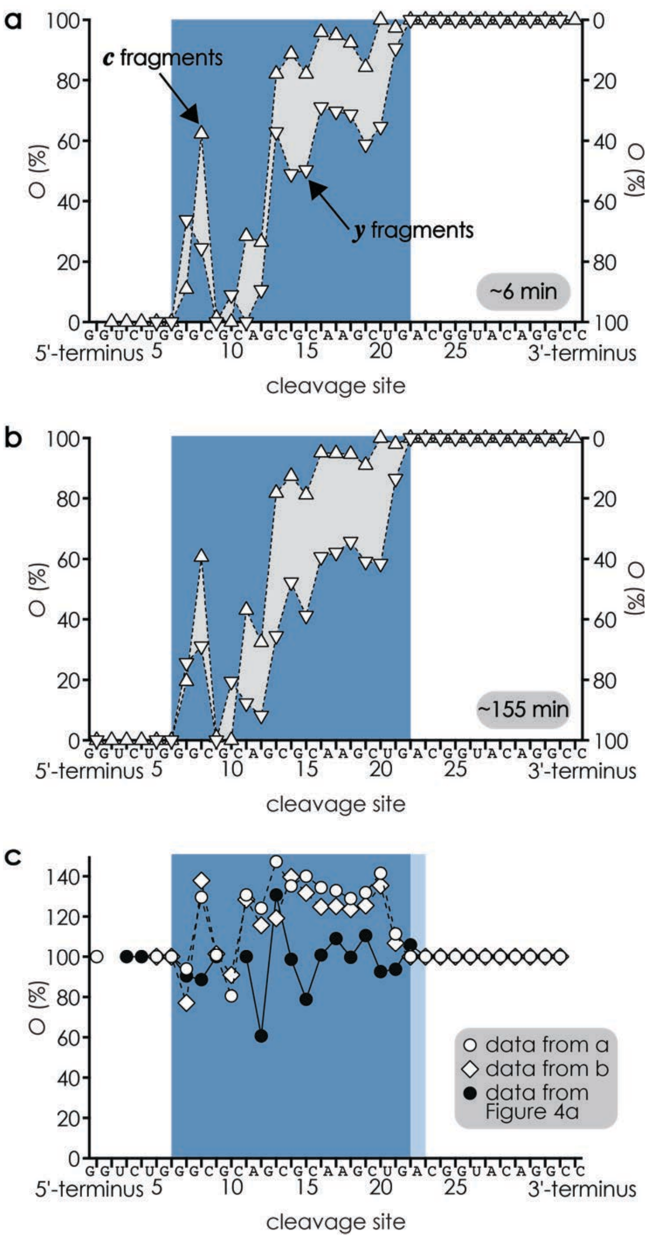

Supplementary Figure 10. Time-resolved binding site mapping of 1:1 complexes. Site-specific occupancy of *c* (upward triangles, left axis) and *y* (downward triangles, right axis) fragments with rev ARM peptide from CAD (137.2 eV) of 1:1 complex ions, (**RRE-TR-0**+rev-14H)$^{14-}$, electrosprayed a) ~6 min and b) ~155 min after preparation of the solutions in 9:1 H_2O/CH_3OH at pH ~7.5 (~1.25 mM piperidine); regions highlighted in blue indicate non-zero occupancy. To minimize the duration of the CAD experiments, fewer scans were recorded at increased ion accumulation time in the collision cell (1.0 s) compared to the experiment in Figure 4a (0.5 s), which generally increased the added non-0% and non-100% occupancy values of *c* and *y* fragments (c) as a result of different stabilities of fragments with (more stable) and without (less stable) peptide attached (kinetic shift) but without affecting the binding region.

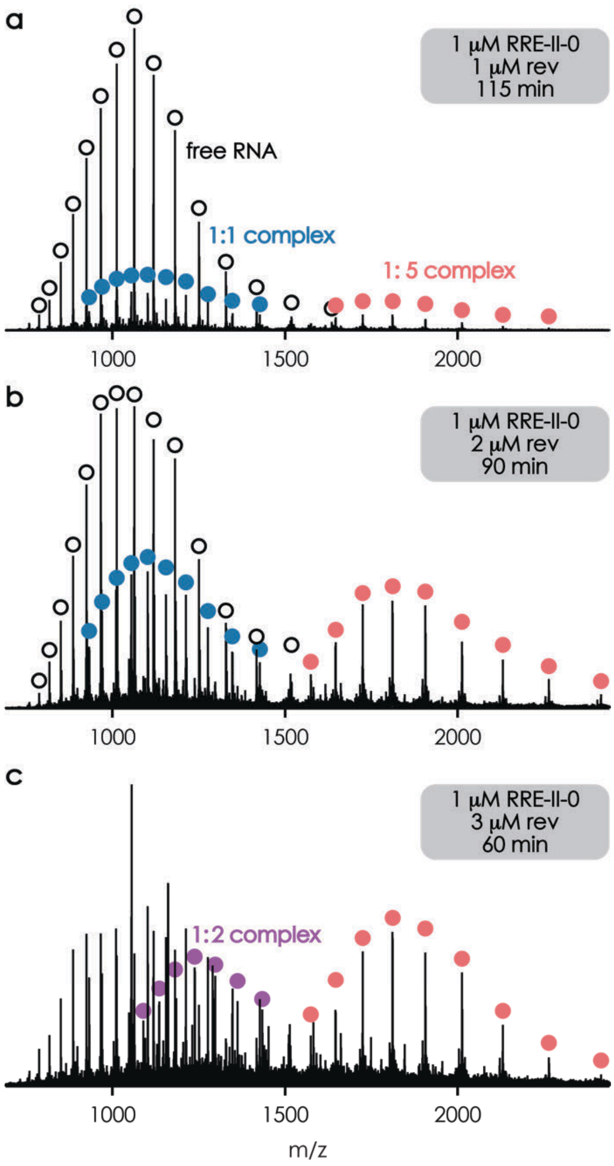

Supplementary Figure 11. Native MS of RRE-II-0. ESI spectra of 1 μM **RRE-II-0** RNA solutions in 9:1 H_2O/CH_3OH with ~1 mM piperidine (pH ~8.1) and a) 1 μM, b) 2 μM, and c) 3 μM rev peptide at ~115, ~90, and ~60 min incubation time, respectively, show signals of free RNA (black circles), 1:1 complex (blue circles), 1:2 complex (violet circles), and 1:5 complex (red circles).

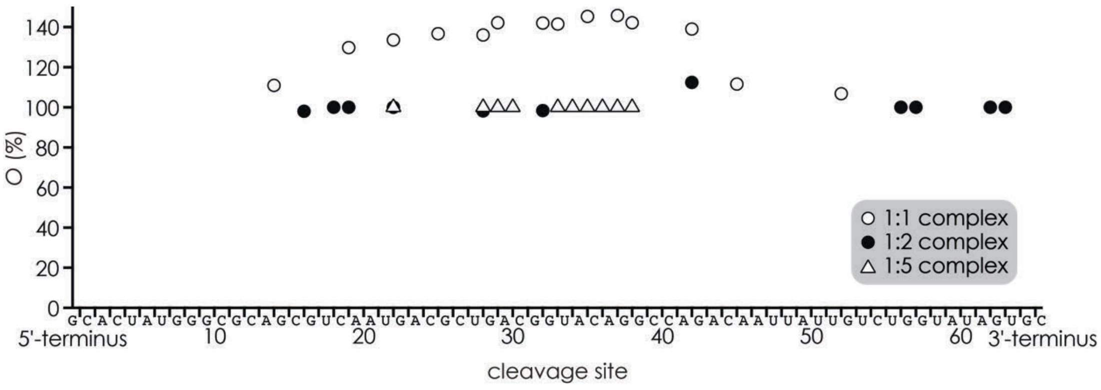

Supplementary Figure 12. Effect of complex stoichiometry on fragment stability. The site-specific, added occupancy values of c and y fragments with rev ARM peptide from CAD of 1:1 (open circles), 1:2 (filled circles), and 1:5 (triangles) complex ions of **RRE-II-0** (data from Figure 6) using 2-3 s ion accumulation time in the collision cell show that differences in the stability of fragments with and without peptide attached (see also Supplementary Figure 10c) decrease with increasing number of peptide molecules in the complexes, from 1:1 to 1:2 to 1:5.

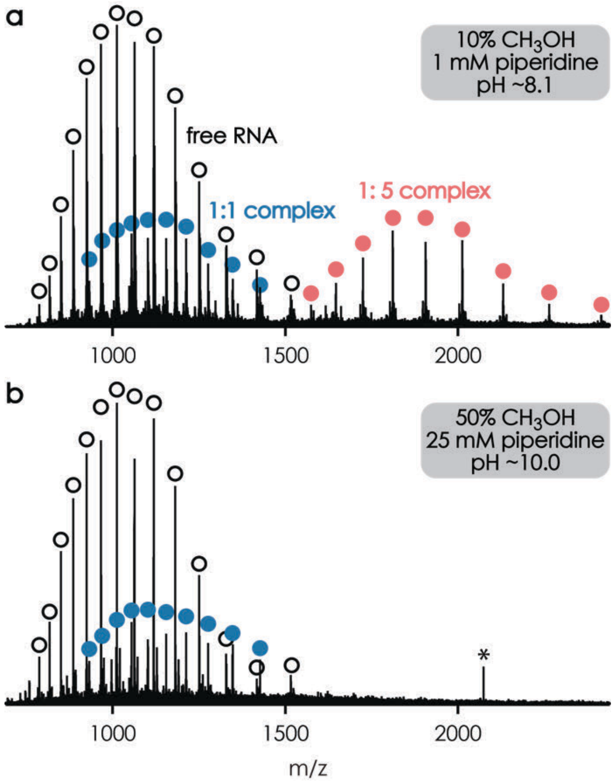

Supplementary Figure 13. Effect of solvent on rev binding. ESI spectra of 1 µM **RRE-II-0** RNA and 2 µM rev peptide solutions in a) 9:1 H_2O/CH_3OH with ~1 mM piperidine (pH ~8.1; this is the same spectrum as in Figure 5b) and b) 1:1 H_2O/CH_3OH with ~25 mM piperidine (pH ~10.0) at ~10 min incubation time both show signals of free RNA (black circles) and 1:1 complex (blue circles), but 1:5 complexes (red circles) were not observed at higher pH and CH_3OH content; the asterisk indicates a signal from radiofrequency interference.
